# Supplementary material for: Gene editing for latent herpes simplex virus infection reduces viral load and shedding in vivo
Source: Nat Commun. 2024 May 13;15:4018. doi: 10.1038/s41467-024-47940-y (PMC11091195; doi:10.1038/s41467-024-47940-y)
Supplement: Supplementary file 1 — Supplementary Information [file 41467_2024_47940_MOESM1_ESM.pdf]

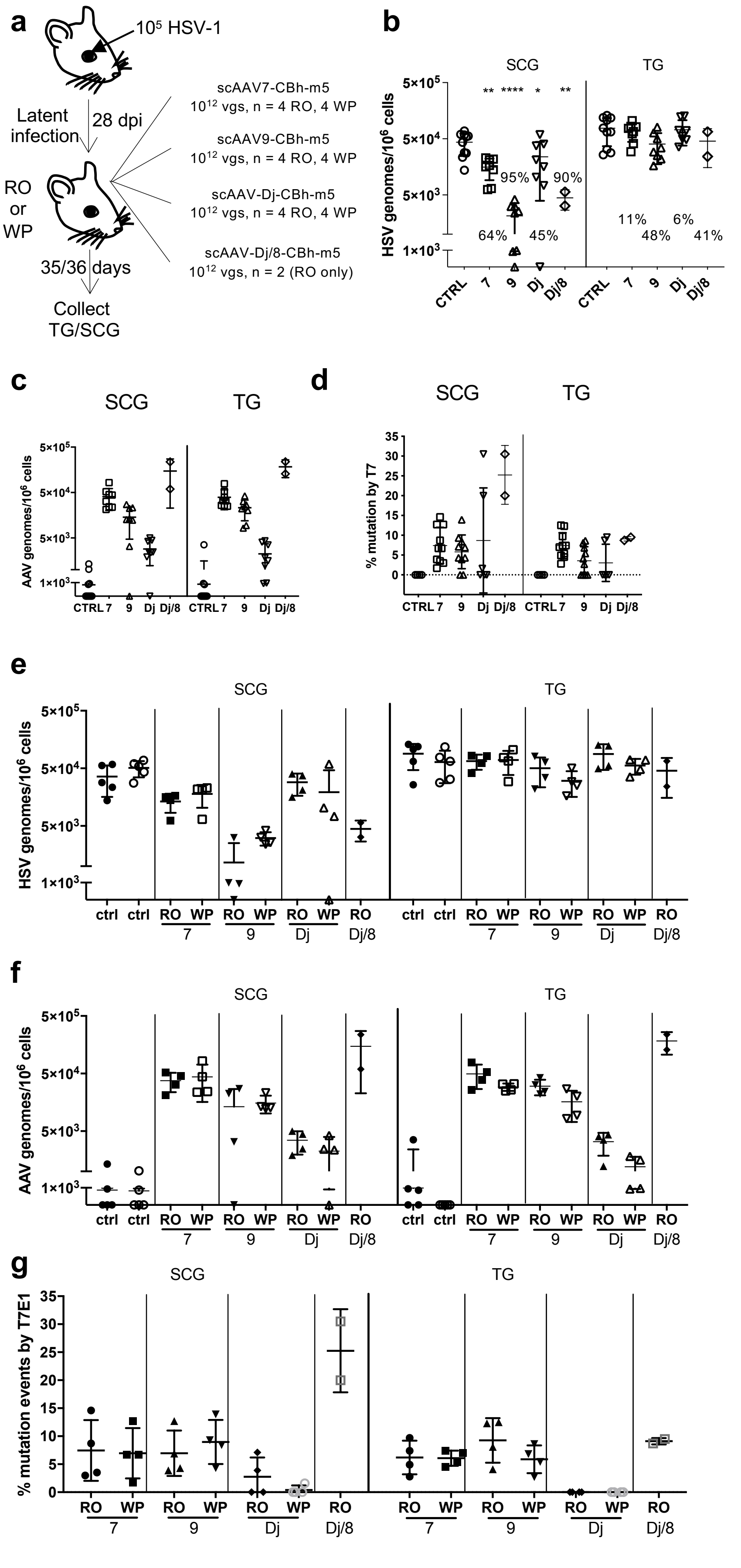

Supplemental Figure 1

Supplemental Figure 1: Reduction of ganglionic latent HSV loads after meganuclease therapy delivered using various AAV serotypes does not depend on the route of administration. **a**. Experimental timeline of ocular infection and meganuclease therapy. **b**. HSV loads in SCGs and TGs from infected control and infected mice treated with m5 delivered by retro-orbital (RO) or whisker pad (WP) injections of 10^{12} vg of various AAV. Percent decrease of HSV loads in treated mice compared to control mice and significant statistical analysis (Ordinary one-way Anova, multiple comparisons with *: $p < 0.05$; **: $p < 0.01$, ****: $p < 0.0001$). Exact p values are provided in the Source Data File. **c**, AAV loads in SCGs and TGs from infected control (CTRL, circles) and infected mice treated with m5 delivered using AAV serotype 7 (squares), 9 (upward triangles), Dj (downward triangles), Dj/8 (diamonds) administered by either RO or WP injections (see Fig. 1a). **d**, Percent mutation quantified by T7 assay in latent HSV genomes present in SCG and TG collected from infected control (CTRL, circles) and infected mice treated with m5 delivered using AAV serotype 7 (squares), 9 (upward triangles), Dj (downward triangles), Dj/8 (diamonds) administered by either RO or WP injections (see Fig. 1a). **e-g**, Same data as above in panels **a-d** presented per route of administration of the AAV delivery vectors. Source data are provided as a Source Data file.

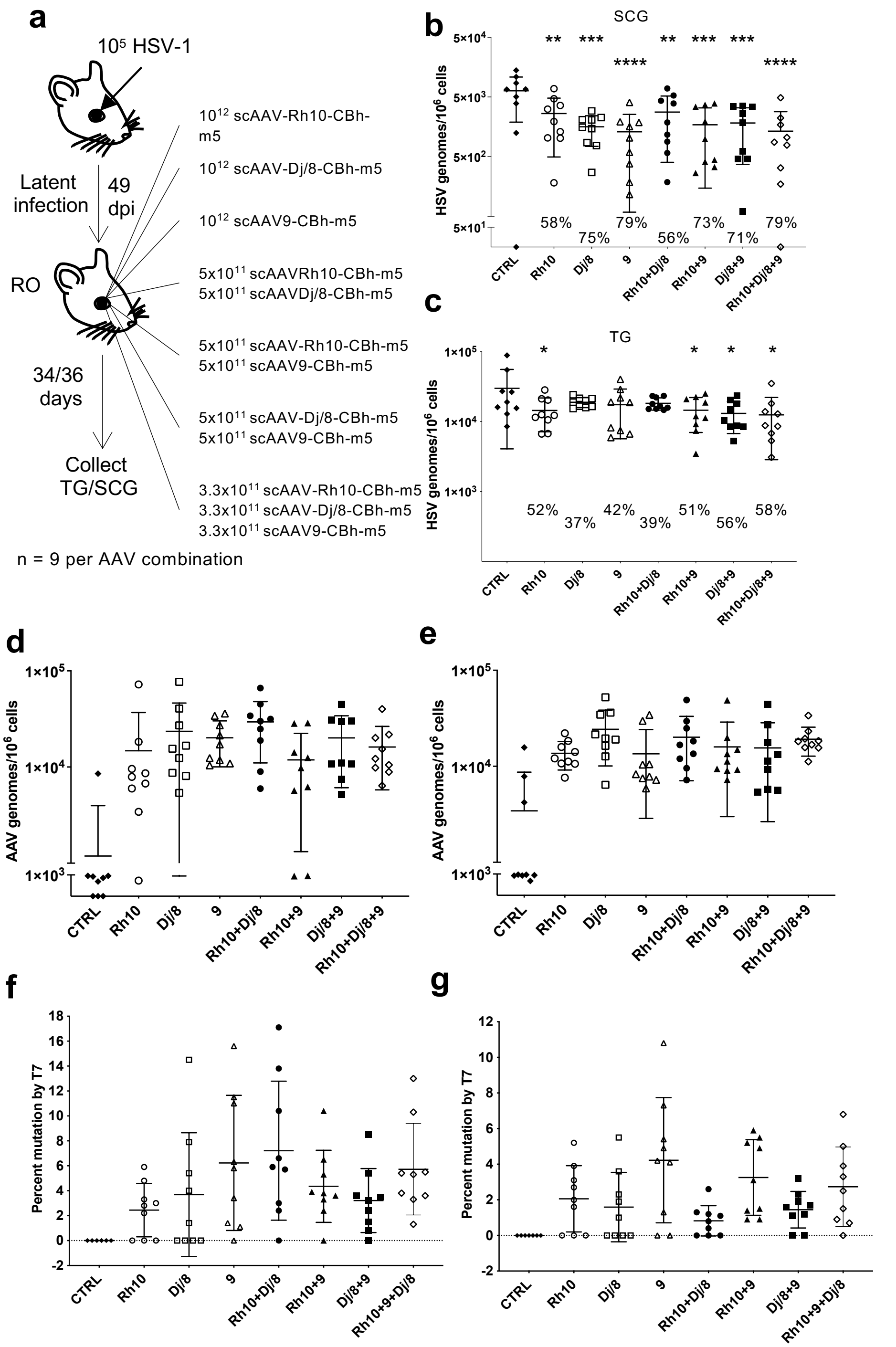

Supplemental Figure 2. Reduction of ganglionic HSV loads after meganuclease therapy delivered using various AAV serotypes combinations. **a,** Experimental timeline of ocular infection and meganuclease therapy. **b-c**. HSV loads in SCGs (**b**) and TGs (**c**) from infected control and infected mice treated with m5 delivered by RO injection of 10^{12} vg of either single AAV serotype, dual AAV serotype combinations or triple AAV serotype combination . Each graph shows individual and mean values with standard deviation, percent decrease of HSV loads and significant statistical difference between treated and control mice (ordinary one-way Anova test with multiple comparisons *: $p < 0.05$; **: $p < 0.01$; ****: $p < 0.0001$). Exact p values are provided in the Source Data File. **d-e**, AAV loads in SCG (**d**) and TG (**e**) from infected control (CTRL, circles) and infected mice treated with m5 delivered using AAV combination of serotype 9, Dj/8 and Rh10 administered by RO injections (see Fig. 1c). **f-g**, Percent mutation quantified by T7 assay in latent HSV genomes present in SCG (**f**) and TG (**g**). Source data are provided as a Source Data file.

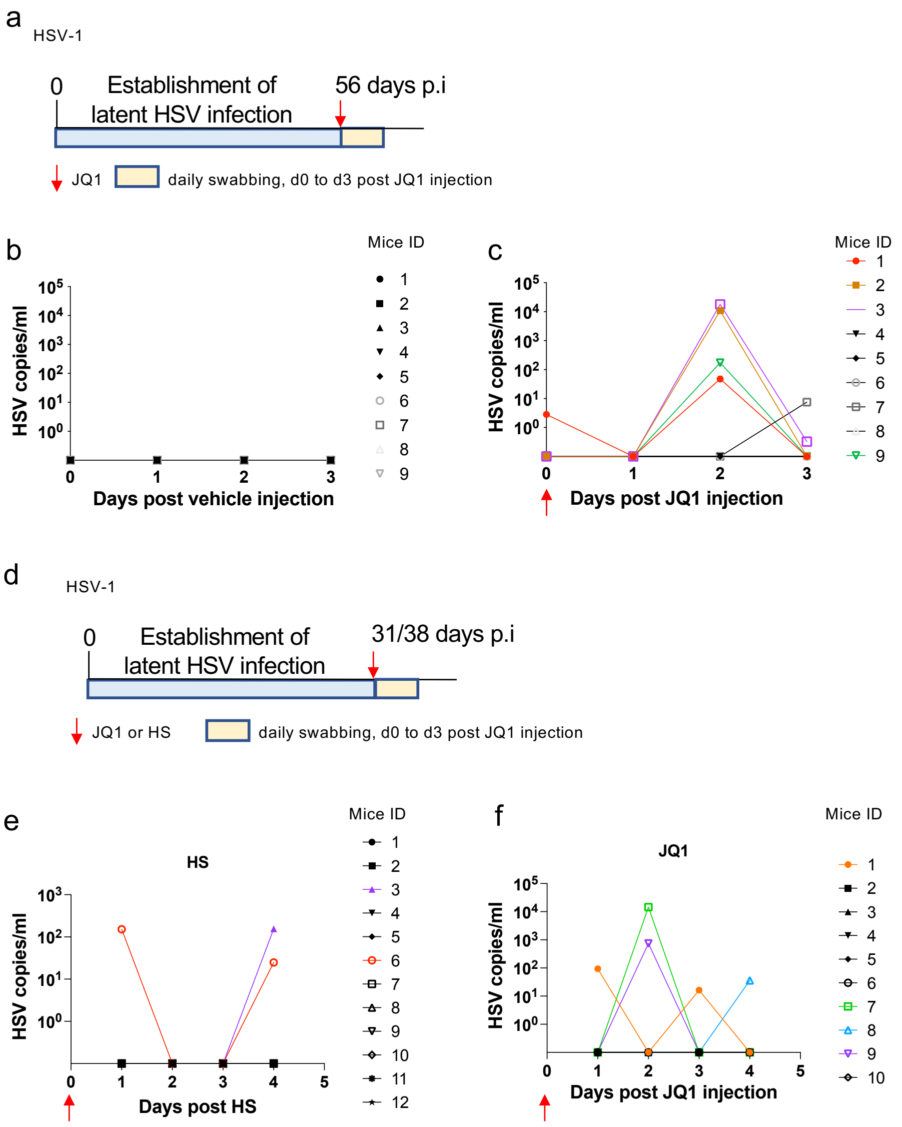

Supplemental Figure 3. JQ1 reactivation leads to peripheral virus shedding. a, Experimental timeline of ocular infection and HSV reactivation. **b-c,** Latently infected mice were administered one IP injection of either (**b**) vehicle n = 9, or (**c**) JQ1 n = 9, red arrow, 50 mg/kg. HSV titers in eye swabs collected from day 0 to 3 post-JQ1. **d**, Experimental timeline of ocular infection and HSV reactivation using hyperthermic stress (31 dpi) or JQ1 injection (38 dpi). **e-f**, Latently infected mice were reactivated at day 0 (red arrow) by either (**e**) hyperthermic stress n = 12, or (**f**) JQ1 IP injection (50 mg/kg) n = 10. HSV titers in eye swabs collected from day 1 to 4 post-JQ1 are plotted for each mouse. Source data are provided as a Source Data file.

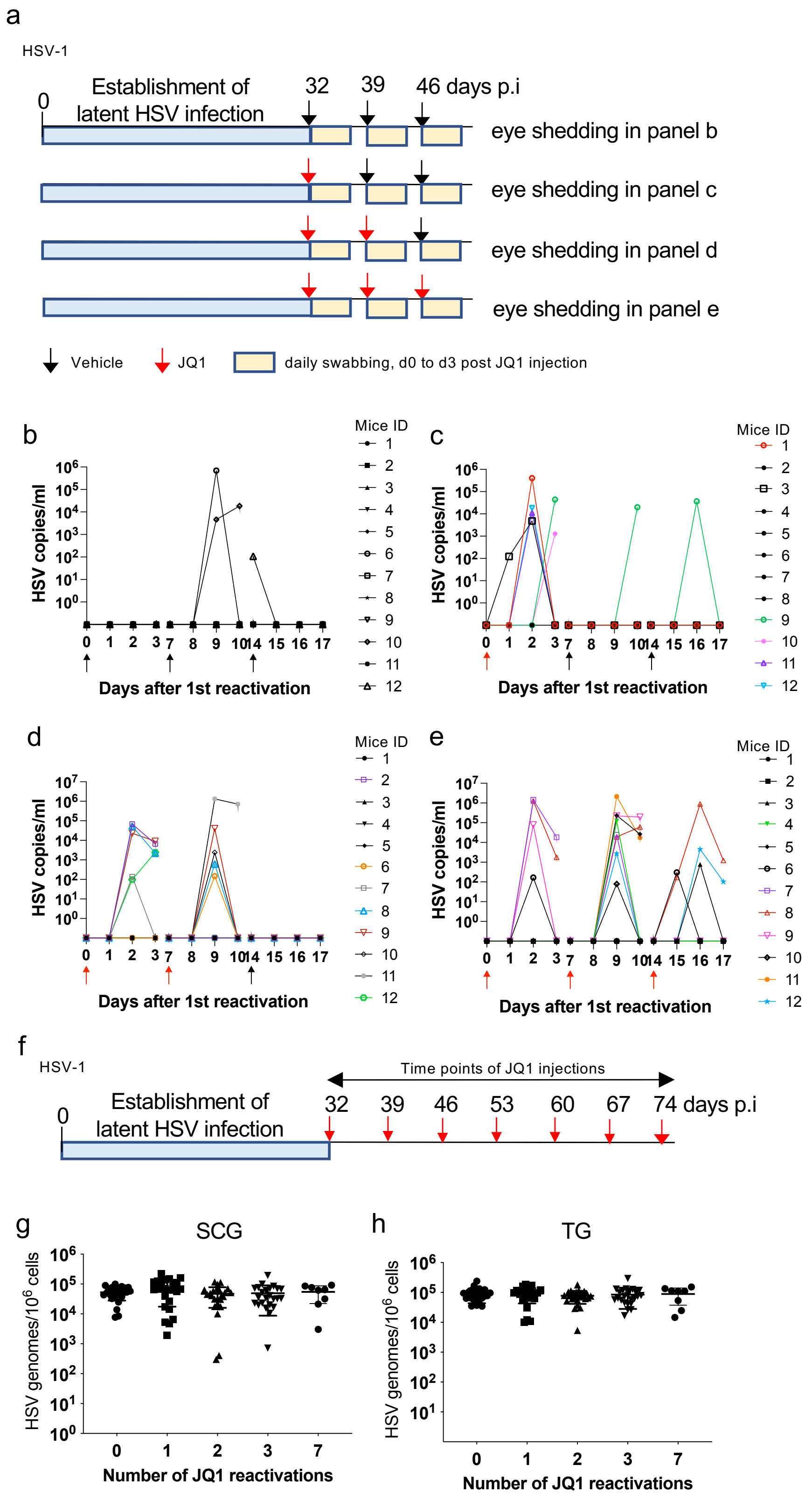

Supplemental Figure 4

Supplemental Figure 4. JQ1 reactivation leads to peripheral virus shedding. a, Experimental timeline of ocular infection and sequential HSV reactivation with JQ1 injections. **b-e,** HSV titers in eye swabs collected daily for 3 days after the 1st (day 32 p.i. in **a** and day 0 in **b-e**), 2nd (day 39 p.i. in **a** and day 7 on graph **b-e**) or 3rd (day 46 p.i. in **a** and day 14 in **b-e**) IP injection of either vehicle (black arrows) or JQ1 (red arrows, 50 mg/kg) **b**, mice (n = 12) received 3 sequential IP injections of vehicle, **c**, mice (n = 12) received 1 IP injection of JQ1 followed by 2 sequential IP injections of vehicle, **d**, mice (n = 12) received 2 sequential IP injections of JQ1 followed by 1 IP injection of vehicle, and **e**, mice received 3 sequential IP injections of JQ1. **f.** Experimental timeline of ocular infection and sequential HSV reactivation with JQ1 injections. **g-h**, HSV viral loads in SCG (**g**) and TG (**h**) collected from mice after either 3 sequential injections of vehicle (0, n = 12), 1 JQ1 injection followed by 2 sequential injections of vehicle (1, n = 12), 2 sequential JQ1 injections followed by 1 injection of vehicle (2, n = 12), 3 sequential JQ1 injections (3, n = 12) or 7 sequential JQ1 injections (7, n = 4). Each graph shows individual and mean values with standard deviation. Source data are provided as a Source Data file.

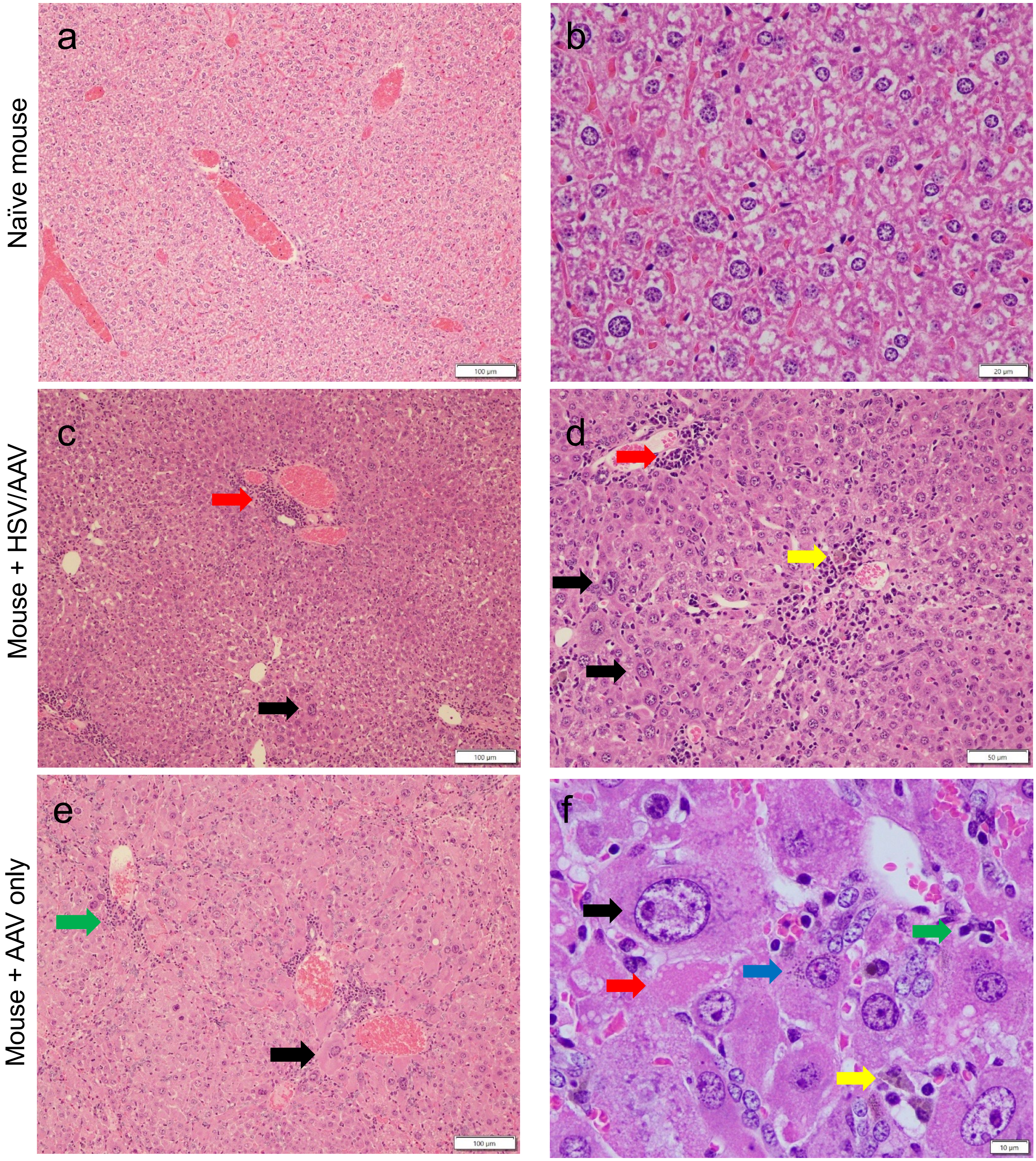

Supplemental figure 5: Histopathology of liver from dual meganuclease-treated mice.
H&E staining of liver section from naive mouse (a,10x and b, 40x), HSV-infected mouse administered 3x10^{12} vg AAV (c, 10x and d, 40x) and mouse administered 3x10^{12} vg AAV only (e, 10x and f, 60x).
Black arrows indicate hepatocellular karyomegaly, anisocytosis and anisokaryosis, red arrows indicate hepatocellular necrosis, green arrows indicate periportal or parenchymal mixed infiltrates, yellow arrow indicates pigmented Kupffer cells, blue arrow indicates suspected biliary cholestasis.

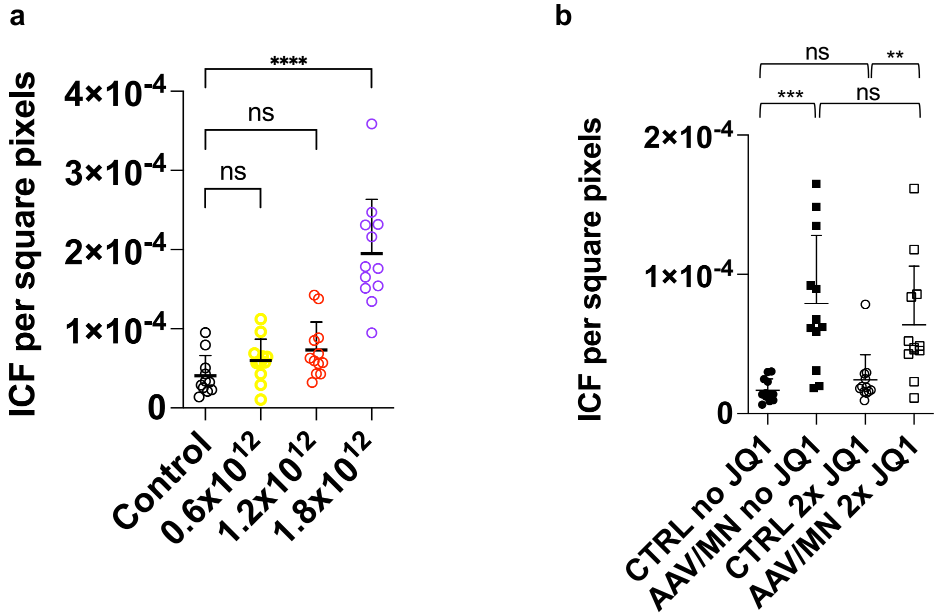

Supplemental figure 6: Inflammatory cell foci in liver of meganuclease-treated mice. a, ICF in liver sections from either HSV infected control mice (control, black circles, n = 11), or treated with dual meganuclease therapy at a dose of $0.6x10^{12}$ vg AAV (yellow circles, n= 12), $1.2x10^{12}$ vg AAV (red circles, n = 12) and $1.8x10^{12}$ vg AAV (purple circles n =12) from experiment described in Figure 5a-l. Statistical analysis using ordinary one-way Anova with multiple comparisons test, ns: not significant; ****: $p < 0.0001$. Exact p values are provided in the Source Data File. **b**, ICF in liver sections from either HSV infected control mice unreactivated (control no JQ1, black circles, n = 12), control mice reactivated with JQ1 (control 2x JQ1, black squares, n =12), HSV infected mice treated with dual meganuclease therapy unreactivated (AAV/MN no JQ1, open circles n =12), or reactivated with JQ1 (AAV/MN 2x JQ1, open squares n = 12) from experiment described in Figure 5m-u. Statistical analysis using unpaired one-tailed *t* test. ns: not significant; **: $p < 0.01$; ***: $p < 0.001$. Exact p values are provided in the Source Data File. Source data are provided as a Source Data file.

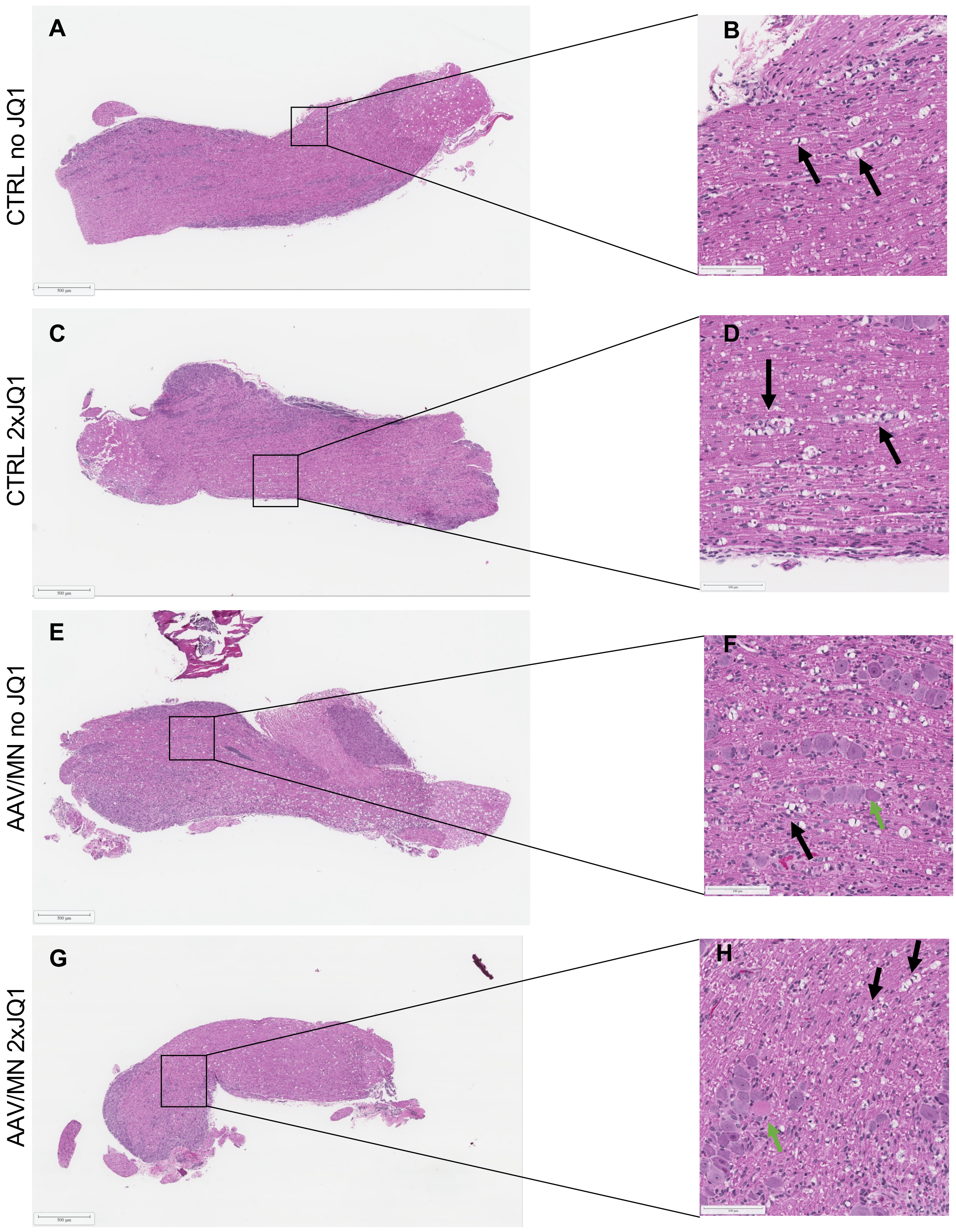

Supplemental figure 7. Histopathology of TG from dual meganuclease-treated mice. Images of H&E stained trigeminal ganglia sections from either latently infected control mice not reactivated (CTRL no JQ1 (slide 10 in Supplemental Table 3): **A**, 2.5x and **B**,20x), and reactivated with JQ1 (CTRL 2xJQ1 (slide 11 in Supplemental Table 3): **C**, 2.5x and **D**, 20x) or $1.8x10^{12}$ vg AAV/dual meganuclease treated mice not reactivated (AAV/MN no JQ1 (slide 1 in Supplemental Table 3): **E**, 2.5x and **F**, 20x) and reactivated (AAV/MN 2xJQ1 (slide 4 in Supplemental Table 3): **G**, 2.5x and **H**, 20x). Black arrows indicate signs of axonopathy, green arrows indicate neurons with central chromatolysis.

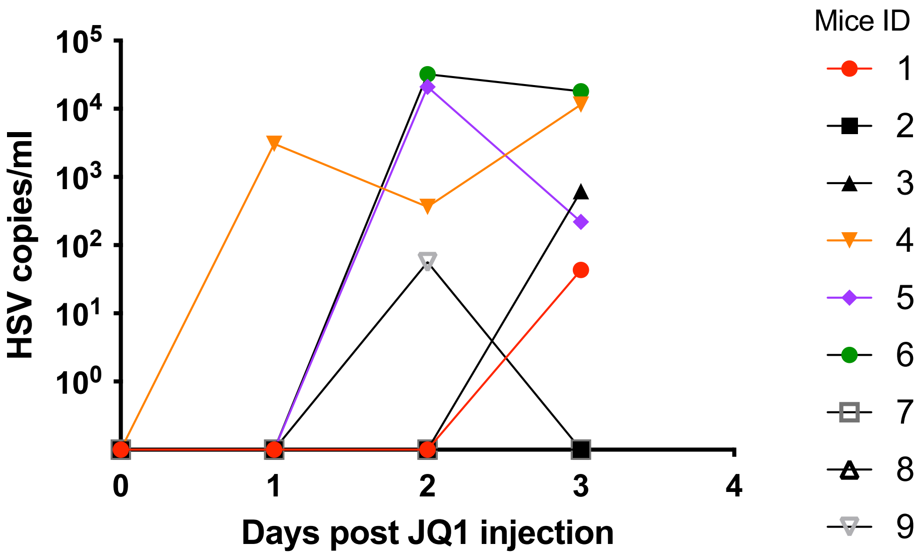

Supplemental Figure 8. Viral shedding after a double dose of JQ1 in 67% (6 /9) of reactivated mice. **a**, Latently infected mice were administered 2 IP injections of JQ1 (50 mg/kg) separated by 12h, n = 9. HSV titers in eye swabs collected from day 0 to 3 post-JQ1 are plotted for each mouse. Source data are provided as a Source Data file.

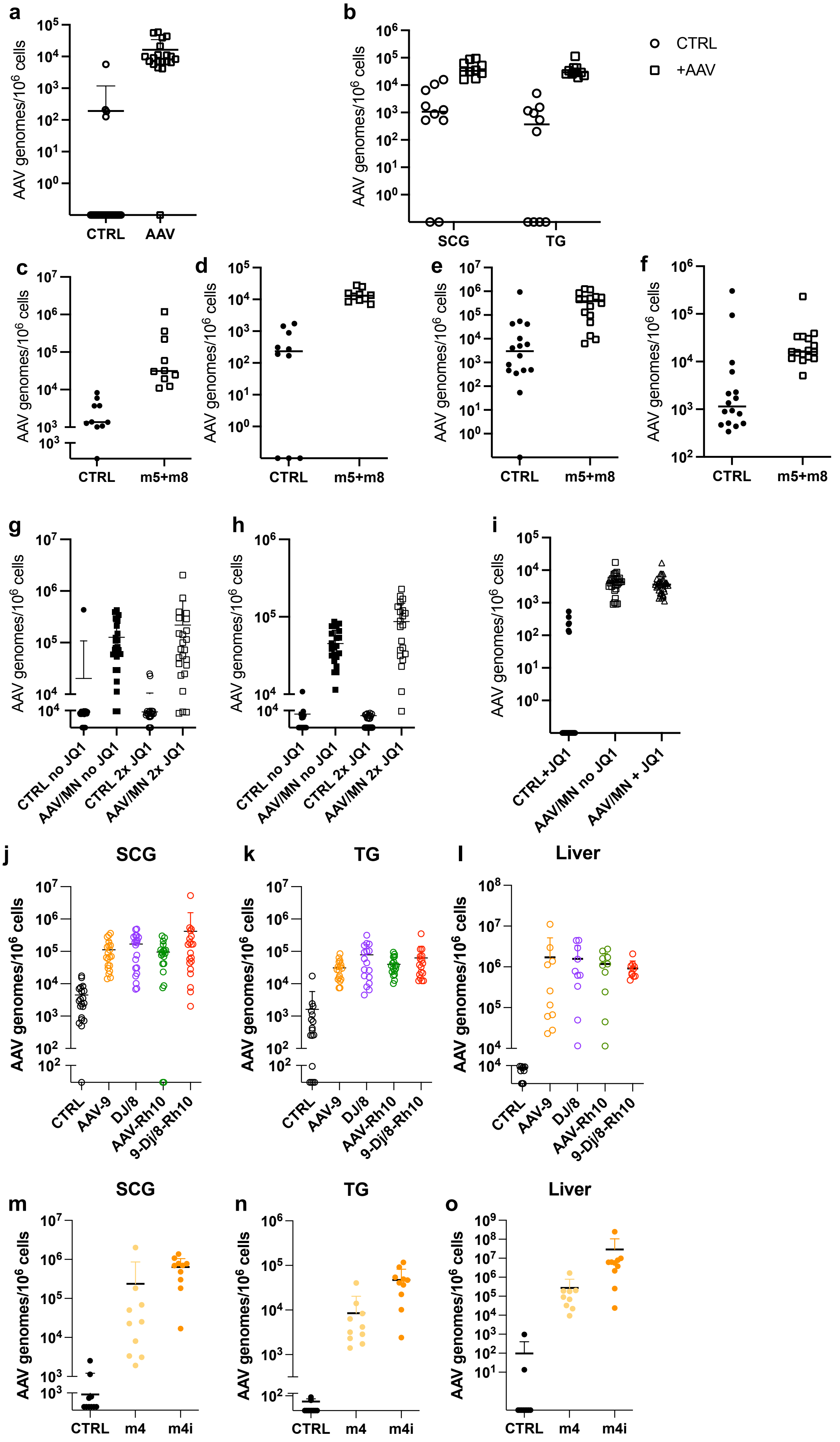

Supplemental Figure 9

Supplemental Figure 9. ddPCR quantification of AAV viral loads. **a**, AAV loads in DRGs from latently infected mice following intravaginal administration of HSV-1 either control untreated (CTRL, open circles, n = 7) or treated with AAV-delivered meganuclease dual therapy (AAV, open squares n = 4) in the experiment presented in Figure 1a, c. **b**, AAV loads in SCG and TG from latently infected mice following ocular administration of HSV-1 either control untreated (CTRL, open circles, n = 10) or treated with AAV-delivered meganuclease dual therapy (AAV, open squares, n = 10) in the experiment presented in Figure 1b, d. **c**-**d**, AAV loads in SCGs (**c**) and TGs (**d**) from latently infected mice following ocular administration of HSV-1 either control untreated (CTRL, circles, n = 10) or treated with AAV-delivered meganuclease dual therapy (AAV, open squares, n = 10) in the experiment presented in Figure 2a-f. **e**-**f**, AAV loads in SCGs (**e**) and TGs (**f**) from latently infected mice following ocular administration of HSV-1 either control untreated (CTRL, circles, n = 8) or treated with AAV-delivered meganuclease dual therapy (AAV, open squares, n = 8) in the experiment presented in Figure 2g-l. **g**-**h**, AAV loads in SCGs (**g**) and TGs (**h**) from latently infected mice following ocular administration of HSV-1 either control untreated no reactivated (CTRL no JQ1, black circles, n =12), treated with AAV-delivered meganuclease dual therapy not reactivated (AAV/MN no JQ1, black squares, n =12), control untreated reactivated twice with JQ1 (CTRL 2x JQ1, circles, n =12) or treated with AAV-delivered meganuclease dual therapy reactivated twice with JQ1 (AAV/MN 2x JQ1, open squares, n =12) in the experiment presented in Figure 4**a**-**g**. **i**, AAV loads in DRGs from latently infected mice following intravaginal administration of HSV-1 either control untreated reactivated with JQ1 (CTRL+JQ1, circles, n = 8), treated with AAV-delivered meganuclease dual therapy not reactivated (AAV/MN no JQ1, open squares, n = 8), or treated with AAV-delivered meganuclease dual therapy reactivated with JQ1 (AAV/MN+JQ1, open triangles, n = 8) in the experiment presented in Figure 4**h**-**k**. **j**-**l**, AAV loads in SCGs (**j**), TGs (**k**) and livers (**l**) from HSV-1 infected control (CTRL, black circles, n= 10) and HSV-1 infected mice treated with m4 delivered with either AAV single serotype 9 (orange circles, n = 10), Dj/8 (purple circles, n= 10), Rh10 (green circles, n= 10), or triple serotypes 9, Dj/8 and Rh10 (red circles, n= 10) administered by either RO in the experiment presented in Figure 5**a**-**g**. **m**-**n**, AAV loads in SCGs (**m**), TGs (**m**) and livers (**0**) from HSV-1 infected control (CTRL, black circles, n= 10) and HSV-1 infected mice treated with either m4 (yellow circles, n= 10), or m4i (orange circles, n= 10) administered by RO in the experiment presented in Figure 6**a**-**g**. Source data are provided as a Source Data file.

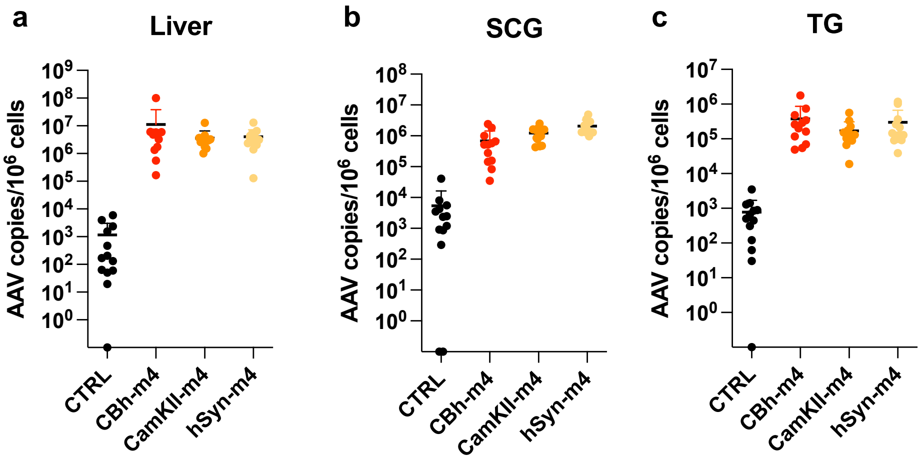

Supplemental Figure 10. ddPCR quantification of AAV viral loads. **a**-**c**l, AAV loads in SCGs (**a**), TGs (**b**) and livers (**c**) from HSV-1 infected control (CTRL, black circles) and HSV-1 infected mice treated with AAV9-CBh-m4 (orange circles), Dj/8 (red circles), AAV9-E/CamKII-m4 (orange circles), or AAV9-E/hSyn-m4 (yellow) administered by either RO in the experiment presented in Figure 7a-f. Source data are provided as a Source Data file.

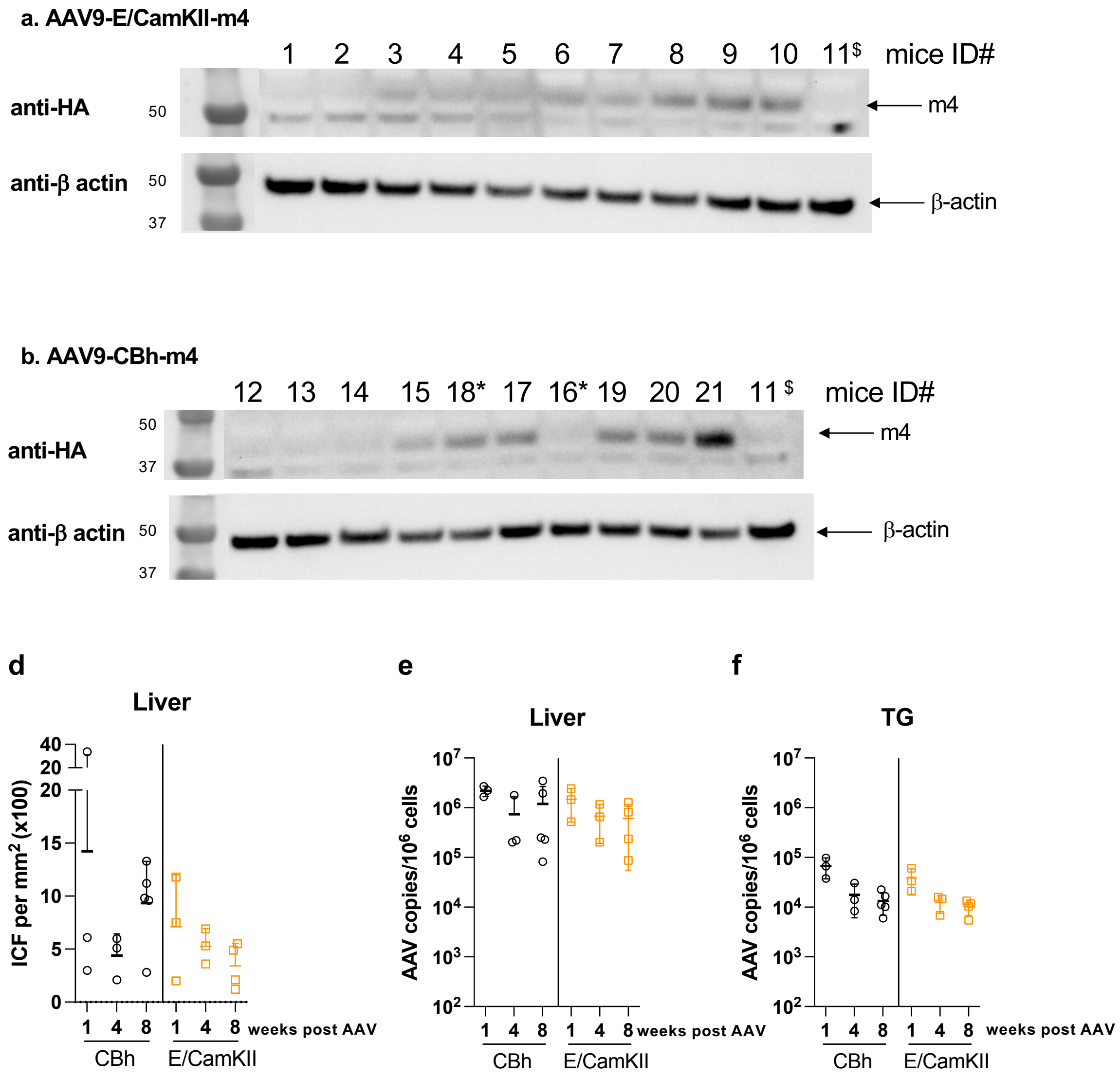

Supplemental Figure 11. Detection of m4 expression in TG from treated mice. **a-b**, Western blot detection of m4 expression (anti-HA) in TG collected from uninfected mice at 1 (mice ID# 1-3 and 12-14) , 4 (mice ID# 4-6 and 15-17), and 8 (mice ID# 7-10, 11, and 18-21) weeks after RO administration of $1x10^{12}$ vg either AAV9-E/CamKII-m4 (**a**), or AAV9-CBh-m4 (**b**). **d**, Immune cell foci (ICF) in liver sections and **e-f**, AAV loads in liver (**e**), and TG (**f**) from uninfected mice treated with either AAV9-CBh-m4 black circles), or AAV9-E/CamKII-m4 (yellow circles) collected at 1, 4, and 8 weeks post AAV administration.

$ Mouse ID#11 did not receive any AAV.

*Sample from mice 16 and 18 are from week 4 and 8, respectively.

The protein molecular weight markers were run on the same gel, but the data was acquired as a separate colorimetric data image from the chemiluminescent data image for the anti-HA (**a**) or anti-β-actin signals (**b**). Source data are provided as a Source Data file.

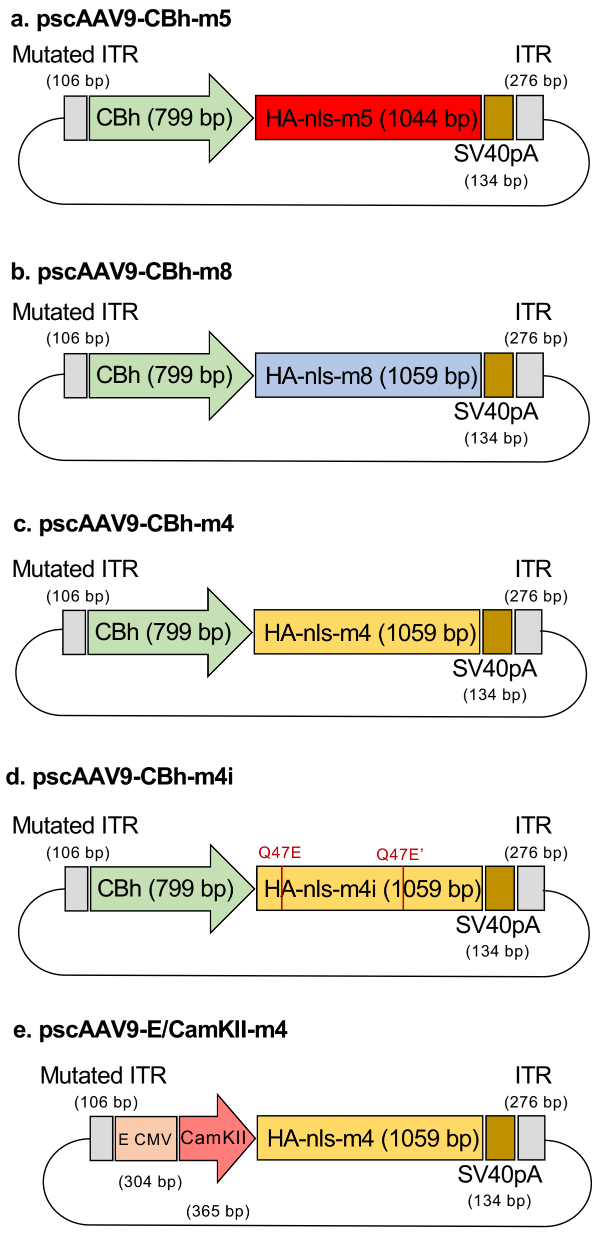

Supplemental Figure 12. Schematics of pscAAV plasmids. Plasmids used for the production of scAAV delivery vectors. ITR: inverted terminal repeat, mutated ITR: ITR with deletion of the D region, HA: HA-tag, nls: nuclear localization signal.

Supplemental Table 1. Virus titers in eye swabs collected after mock and JQ1 reactivations.

Group 1[b]: vehicle - vehicle - vehicle

Mouse	1		2		3		4		5		6		7		8		9		10		11		12	
Day/eye[c]	L	R	L	R	L	R	L	R	L	R	L	R	L	R	L	R	L	R	L	R	L	R	L	R
0	0	0	0	0	0	0	0	0	0	0	0	0	0	0	0	0	0	0	0	0	0	0	0	0
1	0	0	0	0	0	0	0	0	0	0	0	0	0	0	0	0	0	0	0	0	0	0	0	0
2	0	0	0	0	0	0	0	0	0	0	0	0	0	0	0	0	0	0	0	0	0	0	0	0
3	0	0	0	0	0	0	0	0	0	0	0	0	0	0	0	0	0	0	0	0	0	0	0	0
7	0	0	0	0	0	0	0	0	0	0	0	0	0	0	0	0	0	0	0	0	0	0	0	0
8	0	0	0	0	0	0	0	0	0	0	0	0	0	0	0	0	0	0	0	0	0	0	0	0
9	0	0	0	0	0	0	0	0	0	0	0	1379357	0	0	0	0	0	0	0	9240	0	0	0	0
10	0	0	0	0	0	0	0	0	0	0	0	0	0	0	0	0	0	0	0	36992	0	0	0	0
14	0	0	0	0	0	0	0	0	0	0	0	0	0	0	0	0	0	0	0	0	0	0	0	214
15	0	0	0	0	0	0	0	0	0	0	0	0	0	0	0	0	0	0	0	0	0	0	0	0
16	0	0	0	0	0	0	0	0	0	0	0	0	0	0	0	0	0	0	0	0	0	0	0	0
17	0	0	0	0	0	0	0	0	0	0	0	0	0	0	0	0	0	0	0	0	0	0	0	0

Group 2[b]: JQ1 - vehicle - vehicle

Mouse	13		14		15		16		17		18		19		20		21		22		23		24	
Day/eye[c]	L	R	L	R	L	R	L	R	L	R	L	R	L	R	L	R	L	R	L	R	L	R	L	R
0	0	0	0	0	0	0	0	0	0	0	0	0	0	0	0	0	0	0	0	0	0	0	0	0
1	0	0	0	0	245	0	0	0	0	0	0	0	0	0	0	0	0	0	0	0	0	0	0	0
2	0	809002	0	0	9574	0	0	0	0	0	0	0	0	0	0	0	0	0	0	0	21650	0	36754	0
3	0	0	0	0	0	0	0	0	0	0	0	0	0	0	0	0	87982	0	0	2589	0	0	0	0
7	0	0	0	0	0	0	0	0	0	0	0	0	0	0	0	0	0	0	0	0	0	0	0	0
8	0	0	0	0	0	0	0	0	0	0	0	0	0	0	0	0	0	0	0	0	0	0	0	0
9	0	0	0	0	0	0	0	0	0	0	0	0	0	0	0	0	0	0	0	0	0	0	0	0
10	0	0	0	0	0	0	0	0	0	0	0	0	0	0	0	0	39275	655	0	0	0	0	0	0
14	0	0	0	0	0	0	0	0	0	0	0	0	0	0	0	0	0	0	0	0	0	0	0	0
15	0	0	0	0	0	0	0	0	0	0	0	0	0	0	0	0	0	0	0	0	0	0	0	0
16	0	0	0	0	0	0	0	0	0	0	0	0	0	0	0	0	72908	0	0	0	0	0	0	0
17	0	0	0	0	0	0	0	0	0	0	0	0	0	0	0	0	0	0	0	0	0	0	0	0

Group 3[b]: JQ1 - JQ1 - vehicle

Mouse	25		26		27		28		29		30		31		32		33		34		35		36	
Day/eye[c]	L	R	L	R	L	R	L	R	L	R	L	R	L	R	L	R	L	R	L	R	L	R	L	R
0	0	0	0	0	0	0	0	0	0	0	0	0	0	0	0	0	0	0	0	0	0	0	0	0
1	0	0	0	0	0	0	0	0	0	0	0	0	0	0	0	0	0	0	0	0	0	0	0	0
2	0	0	0	138118	0	0	0	0	0	0	0	0	0	277	107615	0	46499	0	0	0	0	0	194	0
3	0	0	0	13196	0	0	0	0	0	0	0	0	0	0	4415	0	18195	0	0	0	0	0	4447	408
7	0	0	0	0	0	0	0	0	0	0	0	0	0	0	0	0	0	0	0	0	0	0	0	0
8	0	0	0	0	0	0	0	0	0	0	0	0	0	0	0	0	0	0	0	0	0	0	0	0
9	0	0	0	0	0	0	0	0	0	0	297	0	0	0	0	1218	0	81523	4809	0	0	2640490	0	0
10	0	0	0	0	0	0	0	0	0	0	0	0	0	0	0	0	0	0	0	0	0	1430093	0	0
14	0	0	0	0	0	0	0	0	0	0	0	0	0	0	0	0	0	0	0	0	0	0	0	0
15	0	0	0	0	0	0	0	0	0	0	0	0	0	0	0	0	0	0	0	0	0	0	0	0
16	0	0	0	0	0	0	0	0	0	0	0	0	0	0	0	0	0	0	0	0	0	0	0	0
17	0	0	0	0	0	0	0	0	0	0	0	0	0	0	0	0	0	0	0	0	0	0	0	0

Group 4[b]: JQ1 - JQ1 - JQ1

Mouse	37		38		39		40		41		42		43		44		45		46		47		48	
Day/eye[c]	L	R	L	R	L	R	L	R	L	R	L	R	L	R	L	R	L	R	L	R	L	R	L	R
0	0	0	0	0	0	0	0	0	0	0	0	0	0	0	0	0	0	0	0	0	0	0	0	0
1	0	0	0	0	0	0	0	0	0	0	0	0	0	0	0	0	0	0	0	0	0	0	0	0
2	0	0	0	0	0	0	0	0	0	0	332	0	1931180	987929	0	2530052	158478	0	0	0	0	0	0	0
3	0	0	0	0	0	0	0	0	0	0	0	0	14642	21781	0	3551	0	0	0	0	0	0	0	0
7	0	0	0	0	0	0	0	0	0	0	0	0	0	0	0	0	0	0	0	0	0	0	0	0
8	0	0	0	0	0	0	0	0	0	0	0	0	0	0	0	0	0	0	0	0	0	0	0	0
9	0	0	0	0	0	0	0	238238	472061	0	0	0	49403	0	194	38237	0	441625	0	157	4275968	0	0	5589
10	0	0	0	0	0	0	0	0	52991	0	0	0	0	0	0	120930	0	392154	0	0	35104	0	0	0
14	0	0	0	0	0	0	0	0	0	0	0	0	0	0	0	0	0	0	0	0	0	0	0	0
15	0	0	0	0	0	0	0	0	0	0	604	0	0	0	343	0	0	0	0	0	0	0	0	0
16	0	0	0	0	0	1541	0	0	0	0	0	0	0	0	1773082	0	0	0	0	0	0	0	0	9005
17	0	0	0	0	0	0	0	0	0	0	0	0	0	0	2442	0	0	0	0	0	0	0	0	208

[a] Virus titers are expressed in copies/ml.

[b] Mice were subjected to 3 weekly reactivation: mock reactivation (vehicle) for Group 1, JQ1 reactivation (50 mg/kg) for Group 4 or combinations of mock (vehicle) and JQ1 (50 mg/kg) reactivations for groups 2 and 3.

[c] At each time point, daily swabs were collected from day 0 to day 3 after each reactivation, from the left eye (L, blue) and right eye (R, orange) and analyzed separately.

Supplemental Table 2. Summary of Histologic Findings in the Liver.

	Naive[c,e]	HSV + AAV/MN[d,e,f]	AAV/MN only[c,e,f]
**No. animals examined**	**2**	**1**	**2**
**Hepatocellular phenotypic alteration**	(0)[a]	(1)	(2)
**Minimal**	-	-	-
**Mild**	-	1[b]	-
**Marked**	-	-	2
**Inflammation mononuclear cell; Periportal and parenchymal**	(0)	(1)	(2)
**Minimal**	-	-	-
**Mild**	-	1	-
**Moderate**	-	-	2
**Hepatocellular necrosis, single cell**	(0)	(1)	(2)
**Minimal**	-	1	-
**Moderate**	-	-	2
**Hyperplasia; Kupffer and oval cell**	(0)	(1)	(2)
**Minimal**	-	-	-
**Moderate**	-	1	2
**Biliary cholestasis**	(0)	(1)	(2)
**Minimal**	-	-	-
**Mild**	-	-	2

[a] Total number of animals with finding.
[b] Number of animals with the indicated severity for a specific finding.
[c] The liver was collected from naïve and AAV/MN only mice at 20 post AAV/MN administration.
[d] The liver was collected from HSV+AAV/MN mice at 33 post AAV/MN administration.
[e] All the mice were age-matched.
[f] AAV/MN was administered at a dose of 3.2 x 10^{12} vg AAV.

Supplemental Table 3. Summary of Histologic Findings in trigeminal ganglia.

	CTRL no JQ1, n = 1[a,b]			CTRL 2xJQ1, n = 1[a,b]			AAV/MN[c] no JQ1, n = 3[a,b]			AAV/MN[c] 2xJQ1, n = 3[a,b]		
**1- Trigeminal ganglia**	Score	Mean score[d]	Mean severity[e]	Score	Mean score[d]	Mean severity[e]	Score	Mean score[d]	Mean severity[e]	Score	Mean score[d]	Mean severity[e]
Inflammation, L/P/E	1	1	1	1	1	1	3 1 1	1.6	1.6	2 1 2	1.6	1.6
Neuronal degeneration	0	0	0	0	0	0	2 0 0	0.7	2	1 0 1	0.7	2
Neuronal necrosis	0	0	0	0	0	0	1 0 1	0.7	1	1 0 1	0.7	2
Perivascular infiltrates	0	0	0	0	0	0	1 0 0	0.3	1	0 0 0	0	0
**Sum - Scores:**	**1**	**1**	**1**	**1**	**1**	**1**	**7 1 2**	**3.3**	**5.6**	**4 1 4**	**3**	**5.6**
**2- Associated nerve fiber**	Score	Mean score[d]	Mean severity[e]	Score	Mean score[d]	Mean severity[e]	Score	Mean score[d]	Mean severity[e]	Score	Mean score[d]	Mean severity[e]
Dilated myelin sheath	2	2	2	2	2	2	3 3 3	3	3	2 1 2	1.7	1.7
Digestion chamber	0	0	0	1	1	1	2 2 0	1.3	2	1 0 1	0.7	1
Perivacular infiltrates	0	0	0	0	0	0	2 1 2	1.7	1.7	1 0 1	0.7	1
Inflammation, L/P/E	1	1	1	1	1	1	1 1 3	1.7	1.7	0 0 0	0	0
**Sum - Scores:**	**3**	**3**	**3**	**4**	**4**	**4**	**8 7 8**	**7.7**	**8.4**	**4 1 4**	**3.1**	**3.7**

[a] Number of TG per slide
[b] Each slide had 3 sections from one TG
[c] AAV dose administered was 1.8 x10^{12} vg/mouse
[d] Mean score = Sum of scores from TG in the group/number of TG in the group
[e] Mean severity = Sum of scores from TG in the group/number of TG with a score > 0 in the group

Supplemental Table 4. JQ1-induced virus shedding in C57BL/6 mice latently infected with HSV-1 17+ (lab strain) or HSV-1 clinical isolates.

HSV Copies/10ul DNA						
**day 0 post JQ1**	**Day 1 post JQ1**	**Day 2 post JQ1**	**Day 3 post JQ1**	**Eye**	**Mouse ID**	**HSV-1 Strain dose**
0	0	0	0	R	1	HSV-1 17+ 2E5 PFU per eye
0	0	0	0	L		
0	0	0	0	R	2	
0	0	0	0	L		
0	0	0	0	R	3	
0	82	279	0	L		
0	0	0	0	R	7	6676 2E5 PFU per eye
0	0	0	0	L		
0	0	0	0	R	8	
0	0	0	0	L		
0	0	0	0	R	9	
0	29	5123	1209	L		
0	0	0	0	R	10	7258 2E5 PFU per eye
0	0	0	0	L		
0	0	0	408	R	11	
0	0	0	0	L		
0	0	34315	0	R	12	
0	0	28	0	L		
0	2	0	0	R	13	7577 2E5 PFU per eye
0	0	0	0	L		
0	0	0	0	R	14	
0	0	1079	0	L		
0	0	0	0	R	15	
3	0	0	0	L		
0	0	0	0	R	16	7261 2E5 PFU per eye
0	0	0	0	L		
0	0	0	0	R	17	
0	0	24	0	L		
0	0	1222	0	R	18	
0	0	0	0	L		

a. anti-HA

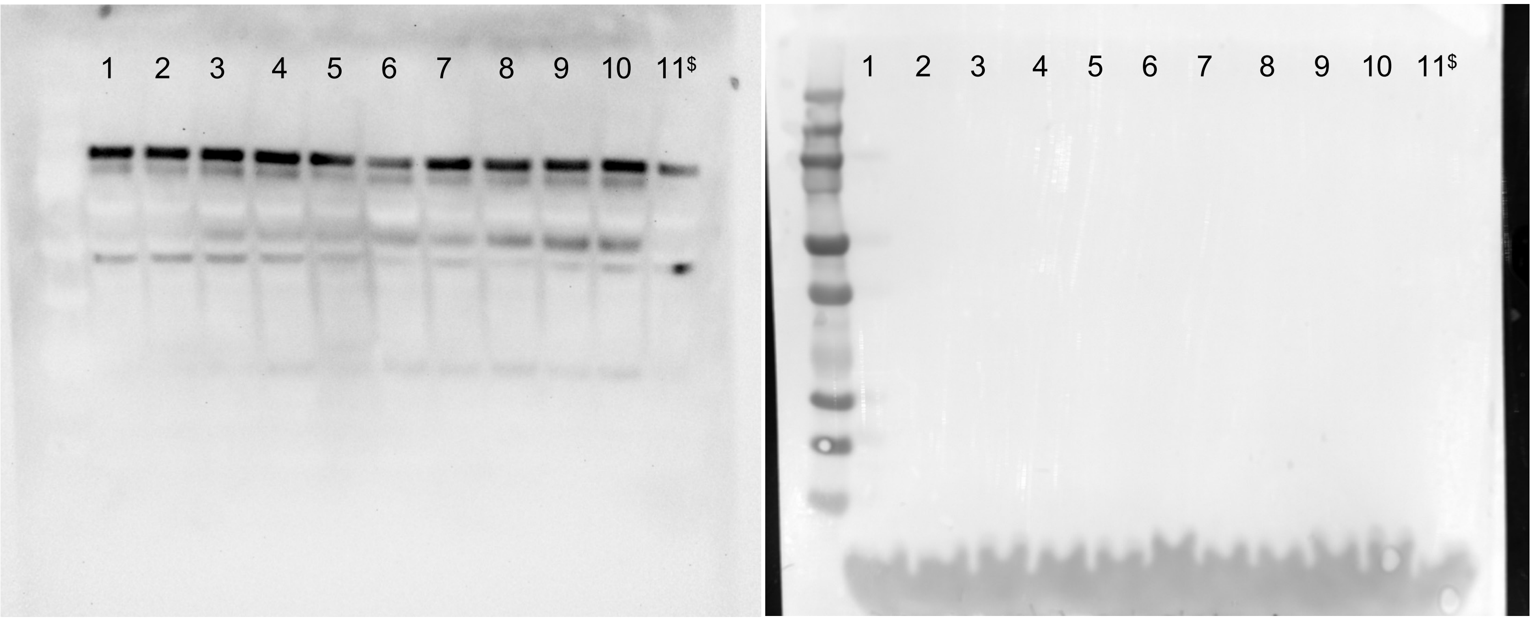

b. anti-β actin

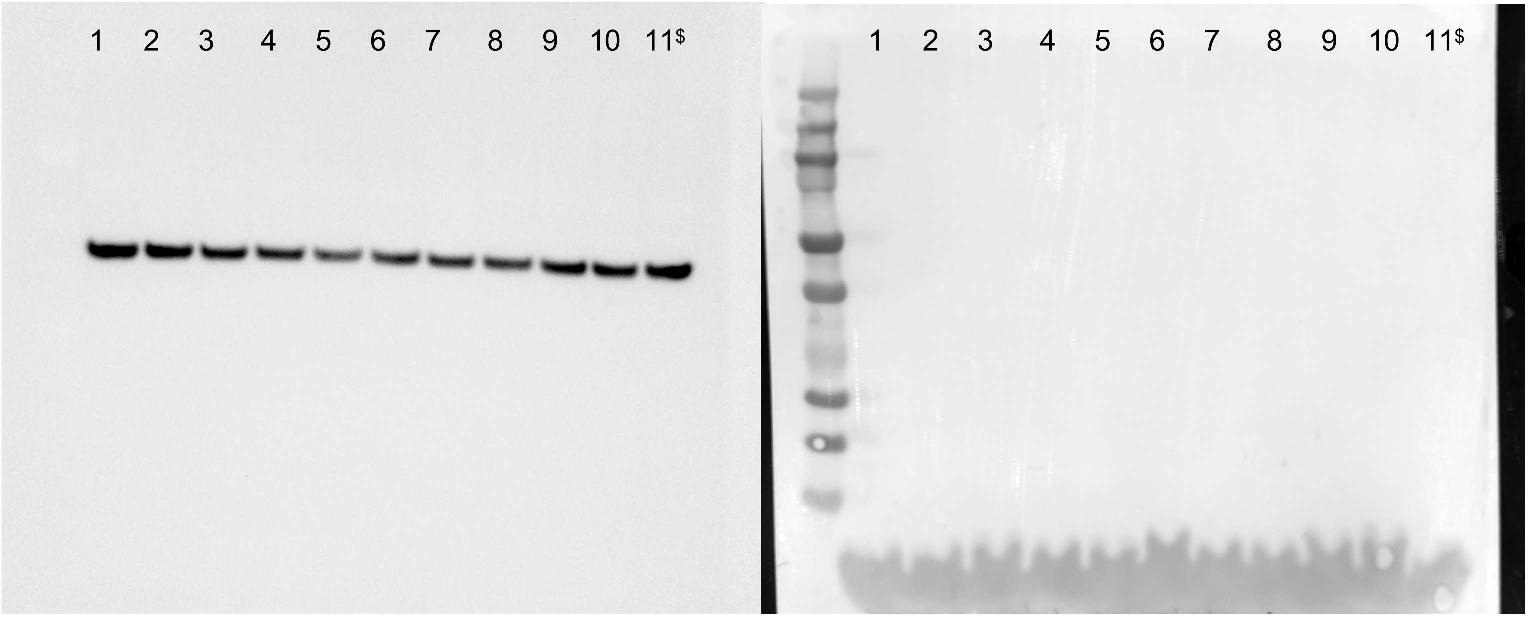

Western blot uncropped scans for Supplemental Figure 11a. Western blot detection of m4 expression (anti-HA) in TG collected from uninfected mice at 1 (mice ID# 1-3) , 4 (mice ID# 4-6), and 8 (mice ID# 7-10, 11) weeks after RO administration of $1x10^{12}$ vg AAV9-E/CamKII-m4

[$] Mouse ID#11 did not receive any AAV.

The membrane was first probed with the anti-HA antibody, then stripped and reprobed with the anti-β-actin antibody.

The protein molecular weight markers were run on the same gel, but the data was acquired as a separate colorimetric data image from the chemiluminescent data image for the anti-HA (**a**) or anti-β-actin signals (**b**).

a. anti-HA

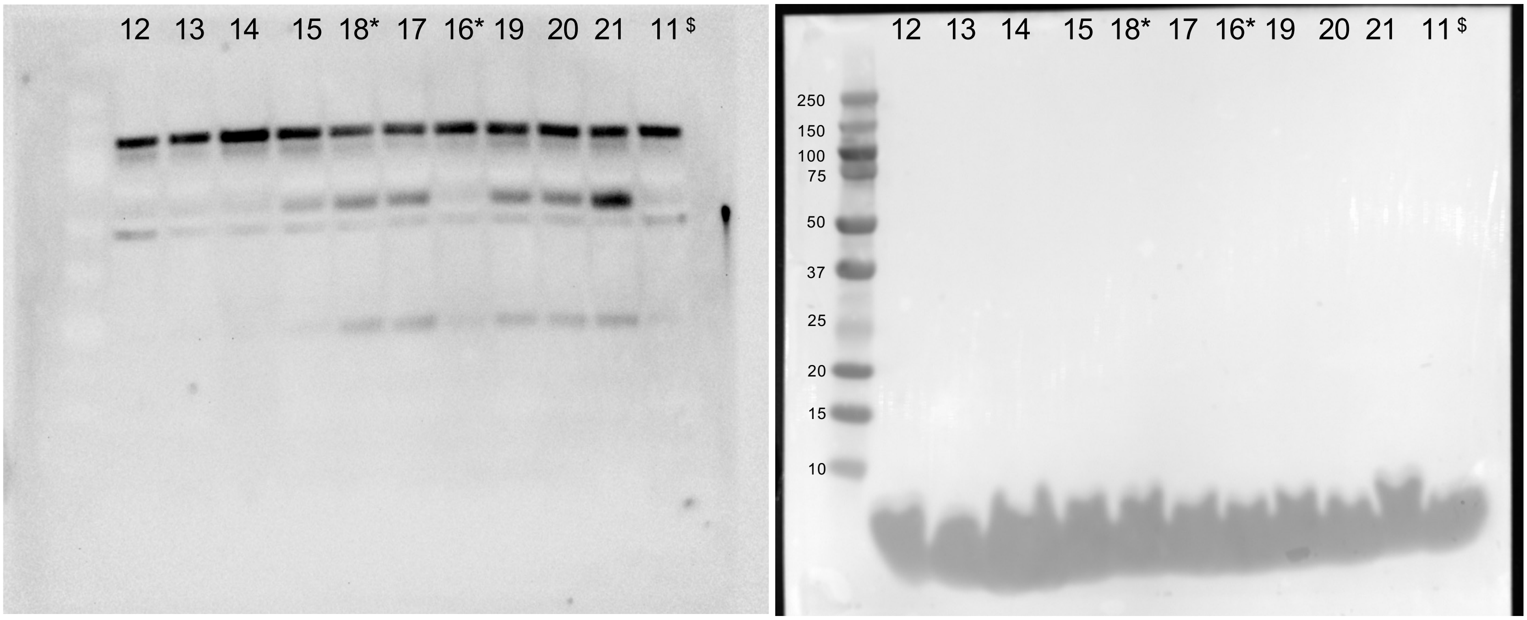

b. anti-β actin

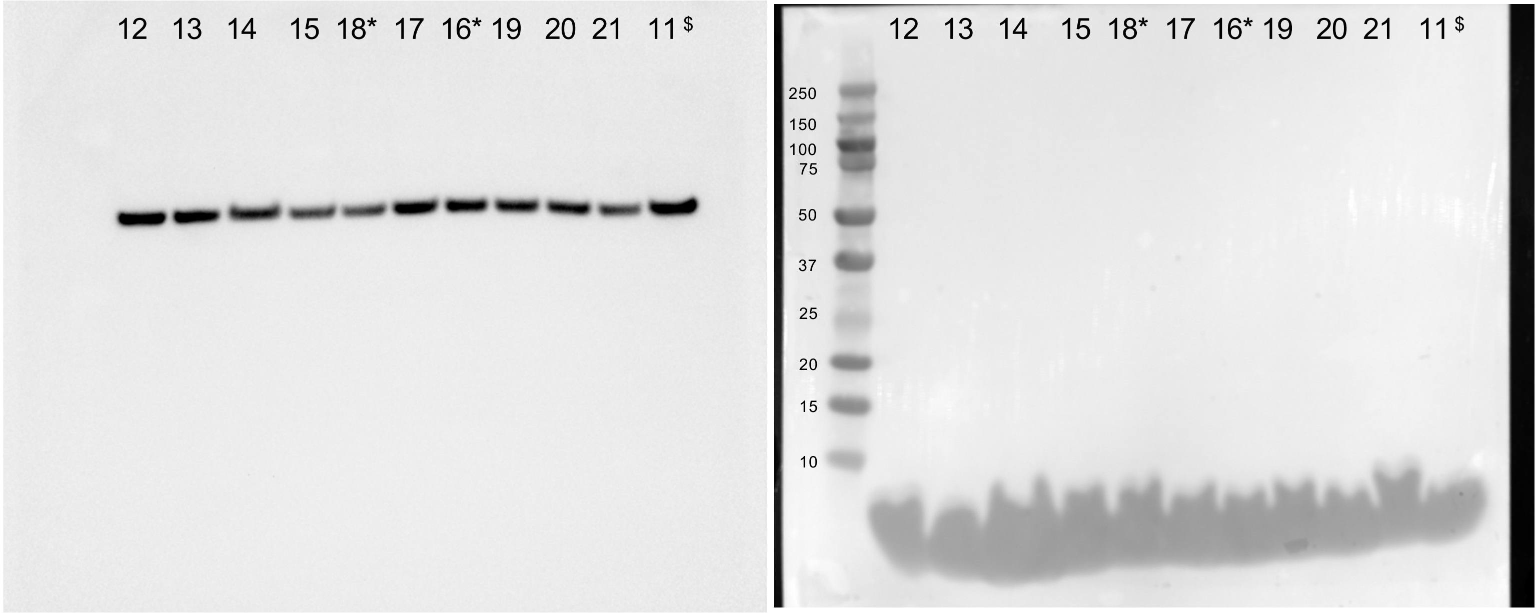

Western blot uncropped scans for Supplemental Figure 11b. Western blot detection of m4 expression (anti-HA) in TG collected from uninfected mice at 1 (mice ID# 12-14) , 4 (mice ID# 15-17), and 8 (mice ID# 11, and 18-21) weeks after RO administration of 1x10^{12} vg AAV9-CBh-m4

[$] Mouse ID#11 did not receive any AAV.

*Sample from mice 16 and 18 are from week 4 and 8, respectively.

The membrane was first probed with the anti-HA antibody, then stripped and reprobed with the anti-β-actin antibody.

The protein molecular weight markers were run on the same gel, but the data was acquired as a separate colorimetric data image from the chemiluminescent data image for the anti-HA (**a**) or anti-β-actin signals (**b**).
