# Supplementary material for: Gene editing for latent herpes simplex virus infection reduces viral load and shedding in vivo
Source: Nat Commun. 2024 May 13;15:4018. doi: 10.1038/s41467-024-47940-y (PMC11091195; doi:10.1038/s41467-024-47940-y)
Supplement: Supplementary file 3 — Reporting Summary [file 41467_2024_47940_MOESM3_ESM.pdf]

nature portfolio

Corresponding author(s): K.R. Jerome

Last updated by author(s): Mar 13, 2024

Reporting Summary

Nature Portfolio wishes to improve the reproducibility of the work that we publish. This form provides structure for consistency and transparency in reporting. For further information on Nature Portfolio policies, see our Editorial Policies and the Editorial Policy Checklist.

Statistics

For all statistical analyses, confirm that the following items are present in the figure legend, table legend, main text, or Methods section.

n/a	Confirmed	
☐	☒	The exact sample size (*n*) for each experimental group/condition, given as a discrete number and unit of measurement
☐	☒	A statement on whether measurements were taken from distinct samples or whether the same sample was measured repeatedly
☐	☒	The statistical test(s) used AND whether they are one- or two-sided<br>*Only common tests should be described solely by name; describe more complex techniques in the Methods section.*
☐	☒	A description of all covariates tested
☐	☒	A description of any assumptions or corrections, such as tests of normality and adjustment for multiple comparisons
☐	☒	A full description of the statistical parameters including central tendency (e.g. means) or other basic estimates (e.g. regression coefficient) AND variation (e.g. standard deviation) or associated estimates of uncertainty (e.g. confidence intervals)
☐	☒	For null hypothesis testing, the test statistic (e.g. F, t, r) with confidence intervals, effect sizes, degrees of freedom and P value noted<br>*Give P values as exact values whenever suitable.*
☒	☐	For Bayesian analysis, information on the choice of priors and Markov chain Monte Carlo settings
☒	☐	For hierarchical and complex designs, identification of the appropriate level for tests and full reporting of outcomes
☒	☐	Estimates of effect sizes (e.g. Cohen's *d*, Pearson's *r*), indicating how they were calculated

Our web collection on statistics for biologists contains articles on many of the points above.

Software and code

Policy information about availability of computer code

Data collection	ddPCR data were collected on BioRad QX200AutoDG Droplet Digital PCR system with QuantaSoft v1.7.4 software.<br>qPCR data were collected on an Applied Biosystem QuantStudio 6 and 7 Flex Real-Time PCR system.<br>Tissue sections images were acquired using an Aperio VERSA 200 digital pathology scanner system.<br>Western blot signals were collected on a BioRad ChemiDoc Touch Imaging system.
Data analysis	Surface area of tissue section were obtained using Fiji.<br>Graphs and statistical analysis were made using GraphPad Prism version 9 and 10.<br>ddPCR data analysis was done using BioRad QX Manager v2.1 sofware and , and GraphPad Prism 9 and 10.<br>qPCR data analysis was done using QuantaSoft version 7, and GraphPad Prism 9 and 10.<br>Histology images were viewed and analyzed using HALO Link Version 3.6.

For manuscripts utilizing custom algorithms or software that are central to the research but not yet described in published literature, software must be made available to editors and reviewers. We strongly encourage code deposition in a community repository (e.g. GitHub). See the Nature Portfolio guidelines for submitting code & software for further information.

Data

Policy information about availability of data

All manuscripts must include a data availability statement. This statement should provide the following information, where applicable:

- Accession codes, unique identifiers, or web links for publicly available datasets
- A description of any restrictions on data availability
- For clinical datasets or third party data, please ensure that the statement adheres to our policy

Data Availability
All data generated are provided in the main text, the supplementary materials or the Source Data file.
Source data is provided with this paper.

Meganuclease sequences are Cellectis proprietary information and material.

Research involving human participants, their data, or biological material

Policy information about studies with human participants or human data. See also policy information about sex, gender (identity/presentation), and sexual orientation and race, ethnicity and racism.

Reporting on sex and gender	N/A
Reporting on race, ethnicity, or other socially relevant groupings	N/A
Population characteristics	N/A
Recruitment	N/A
Ethics oversight	N/A

Note that full information on the approval of the study protocol must also be provided in the manuscript.

Field-specific reporting

Please select the one below that is the best fit for your research. If you are not sure, read the appropriate sections before making your selection.

☒ Life sciences ☐ Behavioural & social sciences ☐ Ecological, evolutionary & environmental sciences

For a reference copy of the document with all sections, see nature.com/documents/nr-reporting-summary-flat.pdf

Life sciences study design

All studies must disclose on these points even when the disclosure is negative.

Sample size	n = 3-5 was chosen for our exploratory experiments, as to limit the animal number used but sufficient to give a meaningful result to identify the optimal parameters.<br>To determine the sample size for each experimental study, we computed the power to detect a difference in viral load at a single time point and calculated that with alpha = 0.05, n = 8-12 mice give the power to detect changes up to 0.5 logs, as seen in preliminary data.
Data exclusions	1-4% of the mice were excluded from the study due to the lack of any acute HSV disease symptoms (ocular infection model) or a day 2 post infection vaginal swab negative for HSV DNA (vaginal infection model); both having been linked to the absence of HSV infection.
Replication	The reproducibility of the findings were verified across the different experiments presented in this study. Each experiment was performed once independently.
Randomization	Mice were allocated to the different treatments based on their acute HSV disease severity score such that each group was comprised of a similar distribution of low, medium and high severity scores.
Blinding	Each collected tissue sample was assigned a reference number upon collection in order to keep investigator blinded during data collection and analysis. Investigators were blinded to treatment group allocations for the recording of data including weight, health, tissue histology analysis, viral load quantifications.

Reporting for specific materials, systems and methods

We require information from authors about some types of materials, experimental systems and methods used in many studies. Here, indicate whether each material, system or method listed is relevant to your study. If you are not sure if a list item applies to your research, read the appropriate section before selecting a response.

Materials & experimental systems

n/a	Involved in the study
☐	☒ Antibodies
☐	☒ Eukaryotic cell lines
☒	☐ Palaeontology and archaeology
☐	☒ Animals and other organisms
☒	☐ Clinical data
☒	☐ Dual use research of concern
☒	☐ Plants

Methods

n/a	Involved in the study
☒	☐ ChIP-seq
☒	☐ Flow cytometry
☒	☐ MRI-based neuroimaging

Antibodies

Antibodies used

Rabbit anti-HA monoclonal antibody (1:1000), clone C29F4 from Cell signaling Catalogue # 3724 and rabbit anti-b-actin monoclonal antibody (1:1000), clone 13E5 from Cell signaling Catalogue # 4970.

Validation

Manufacturer's validation statements:
HA-Tag (C29F4) Rabbit mAb
Product Usage Information
For optimal ChIP results, use 10 µl of antibody and 10 µg of chromatin (approximately 4 x 106 cells) per IP. This antibody has been validated using SimpleChIP® Enzymatic Chromatin IP Kits.
Application Dilution
Western Blotting 1:1000
Simple Western™ 1:50 - 1:250
Immunoprecipitation 1:50
Immunohistochemistry (Paraffin) 1:800 - 1:3200
Immunofluorescence (Immunocytochemistry) 1:800 - 1:1600
Flow Cytometry (Fixed/Permeabilized) 1:800 - 1:1600
Chromatin IP 1:50
Specificity / Sensitivity
HA-Tag (C29F4) Rabbit mAb detects exogenously expressed proteins containing the HA epitope tag. The antibody may cross-react with a protein of unknown origin ~100kDa.
Species Reactivity:
All Species Expected: H-Human M-Mouse R-Rat Hm-Hamster Mk-Monkey Vir-Virus Mi-Mink C-Chicken Dm-D. melanogaster X-Xenopus Z-Zebrafish B-Bovine Dg-Dog Pg-Pig Sc-S. cerevisiae Ce-C. elegans Hr-Horse GP-Guinea Pig Rab-Rabbit
Source / Purification
Monoclonal antibody is produced by immunizing animals with a synthetic peptide containing the influenza hemagglutinin epitope (YPYDVPDYA). The HA tag is derived from an epitope of the influenza hemagglutinin protein which has been used extensively as a general epitope tag in expression vectors (1).

(1) Field, J. et al. (1988) Mol Cell Biol 8, 2159-65.

β-Actin (13E5) Rabbit mAb
Application Dilution
Western Blotting 1:1000
Simple Western™ 1:10 - 1:50
Immunohistochemistry (Paraffin) 1:50 - 1:200
Immunofluorescence (Frozen) 1:100 - 1:400
Immunofluorescence (Immunocytochemistry) 1:100 - 1:400
Flow Cytometry (Fixed/Permeabilized) 1:100 - 1:400
Specificity / Sensitivity
β-Actin (13E5) Rabbit mAb detects endogenous levels of total β-actin protein. Depsite the high sequence identity between the cytoplasmic actin isoforms, β-actin and cytoplasmic γ-actin, β-Actin (13E5) Rabbit mAb #4970 does not cross-react with cytoplasmic γ-actin, or any other actin isoforms.
Species Reactivity:
Human, Mouse, Rat, Monkey, Bovine, Pig
Species predicted to react based on 100% sequence homology:
Hamster, Chicken, Dog, Horse, Rabbit
Source / Purification
Monoclonal antibody is produced by immunizing animals with a synthetic peptide corresponding to residues near the amino-terminus of human β-actin protein.

Eukaryotic cell lines

Policy information about cell lines and Sex and Gender in Research

Cell line source(s)	HEK293 was obtained from Senator P. Wellstone UW vector core facility.<br>Vero cell lines (ATCC # CCL-81) was originally obtained for ATCC
Authentication	None of the cell lines used were authenticated
Mycoplasma contamination	The cell lines were not tested for mycoplasma contamination
Commonly misidentified lines<br>(See ICLAC register)	no misidentified cell lines were used

Animals and other research organisms

Policy information about studies involving animals; ARRIVE guidelines recommended for reporting animal research, and Sex and Gender in Research

Laboratory animals	Mus musculus, strain Swiss Webster, or C57BL/6 5-8 weeks old
Wild animals	The study did not involved wild animals
Reporting on sex	Female mice were used for these studies as vaginal infection can only apply to this sex.
Field-collected samples	The study did not involved samples collected from the field
Ethics oversight	Animals were housed in accordance with the Fred Hutch Cancer Center and NIH guidelines on the care and use of animals in research. Experimental procedures performed and approved by the Institutional Animal Care and Use Committee (IACUC) of the Fred Hutch Cancer Center.

Note that full information on the approval of the study protocol must also be provided in the manuscript.

Plants

Seed stocks	N/A
Novel plant genotypes	N/A
Authentication	N/A
